# Death receptor 6 promotes ovarian cancer cell migration through KIF11

**DOI:** 10.1002/2211-5463.12492

**Published:** 2018-08-07

**Authors:** Bianhua Shi, Jiayu Bao, Yongbin Liu, Juan Shi

**Affiliations:** ^1^ National Laboratory of Medical Molecular Biology Institute of Basic Medical Sciences Chinese Academy of Medical Sciences & Peking Union Medical College Beijing China

**Keywords:** death receptor 6, DR6, KIF11, migration, ovarian cancer, TRAF4

## Abstract

The expression of death receptor 6 (DR6) is abnormal in some cancer types, but the function and underlying molecular mechanisms of DR6 in tumor progression are not yet clear. In the present study, our analysis of ovarian cancer RNA sequencing data from The Cancer Genome Atlas revealed that DR6 is upregulated in human ovarian cancer. We confirmed that the expression level of DR6 is upregulated in ovarian cancer tissues when compared with matched adjacent normal tissues. In addition, DR6 enhanced ovarian carcinoma cell migration ability, and decreased expression of DR6 inhibited the expression of matrix metalloprotease (MMP) 2 and MMP9, and increased the expression of E‐cadherin. Additionally, DR6 shRNA caused a significant decrease in phosphoinositide‐3‐kinase (PI3K), phospho (p) ‐AKT, p‐extracellular signal‐regulated kinase (ERK), and p‐mitogen‐activated protein kinase kinase expression in SKOV3 cells. These results suggested that DR6 can enhance ovarian carcinoma cell migration ability through the mitogen‐activated protein kinase/ERK and PI3K/AKT pathways. Notably, mass spectrometric analysis indicated that DR6 co‐purified with kinesin family member 11 (KIF11), and we verified the interaction between KIF11 and DR6 by co‐immunoprecipitation and glutathione *S*‐transferase pull‐down. Furthermore, we demonstrated that DR6 can bind tumor necrosis factor receptor‐associated factor 4 (TRAF4) with co‐immunoprecipitation. Overexpression of KIF11 or TRAF4 eliminated the suppression of carcinoma cell migration by DR6 knockdown. We also found that TRAF4 and KIF11 were upregulated in ovarian carcinomas and that their level of expression was positively correlated with that of DR6. The findings above suggest that DR6 may play a notable oncogenic role in ovarian malignancy by interacting with TRAF4 and KIF11, and that DR6 may be an effective therapeutic target in ovarian cancer.

**Abbreviations**
DR6: death receptor 6
ERK: extracellular signal‐regulated kinase
GAPDH: glyceraldehyde 3‐phosphate dehydrogenase
GST: glutathione *S*‐transferase
HOSE: human ovarian surface epithelial cells
IB: immunoblotting
IP: immunoprecipitation
KIF11: kinesin family member 11
MAPK: mitogen‐activated protein kinase
MEK: mitogen‐activated protein kinase kinase
MMP: matrix metalloprotease
MTS: 3‐(4,5‐dimethylthiazol‐2‐yl)‐5‐(3‐carboxymethoxyphenyl)‐2‐(4‐sulfophenyl)‐2*H*‐tetrazolium
MS: mass spectrometry
OVCA: ovarian cancer
p: phospho
PI3K: phosphoinositide‐3‐kinase
PVDF: poly(vinylidene) difluoride
shRNA: short hairpin RNA
siRNA: small interfering RNA
TCGA: The Cancer Genome Atlas
TNF: tumor necrosis factor
TRADD: tumor necrosis factor receptor‐associated death domain protein
TRAF4: tumor necrosis factor receptor‐associated factor 4

Death receptor 6 (DR6) is a member of the tumor necrosis factor (TNF) receptor superfamily 1. The specific ligands activate death receptors to mediate diverse biological processes, including cell apoptosis and immune response 2, 3, 4. In the neuronal system, brain vascular formation is suppressed in DR6^−/−^ mice and in zebrafish 5, and DR6 deficiency has been identified as promoting remyelination in a demyelination model 6.

As a death receptor, DR6 is reported to be involved in apoptotic cell death. The cleaved N‐terminal fragment of β‐amyloid precursor protein binds to DR6 to trigger axon pruning and neuron death via caspase 6 in the central nervous system 7, while the antagonist antibody of DR6 promotes motor neuron survival 8. DR6 can bind TNF receptor‐associated death domain protein (TRADD) when overexpressed 1. Furthermore, the agonistic antibody for DR6 activates downstream signaling involved in the recruitment of TRADD 9.

DR6 is highly expressed in certain cancer types; however, less is known about its role in tumor progression. Thus, the function of DR6 in cancer needs to be clarified. The expression of anti‐apoptosis molecules is elevated due to DR6 overexpression in certain cancer cells 10. It has been found that the expression of DR6 in serum is enhanced in patients with late‐stage ovarian cancer (OVCA) 11. Additionally, tumor‐cell‐induced endothelial cell necroptosis occurs via DR6 12; however, the function of DR6 in cancer cells, particularly in OVCA, still requires investigation. To this end, in the current study, we report that DR6 plays a vital oncogenic role in promoting ovarian cell migration by interacting with kinesin family member 11 (KIF11) and TNF receptor‐associated factor 4 (TRAF4), and that DR6 may be an effective therapeutic target in OVCA.

## Materials and methods

### Cell lines and reagents

Cell Culture Center, Chinese Academy of Medical Sciences (Beijing, China) provided the OVCA cell lines SKOV3, HO‐8910, A2780 and OVCAR3. Cells were maintained at 37 °C with 5% CO_2_ in RPMI 1640 (Invitrogen, Carlsbad, CA, USA) with 10% fetal bovine serum (FBS; Invitrogen). Cells were transiently transfected with plasmid or small interfering RNAs (siRNAs) against KIF11 or TRAF4 (Table 1, GenePharma, Shanghai, China) using Lipofectamine^®^ 2000 (Invitrogen) according to the manufacturer's instructions. Target proteins expression was checked by western blotting at 48 h after transfection.

**Table 1:** Primers and siRNA sequence

Primer and siRNA	Sequence
GAPDH forward	5′‐CGGATTTGGTCGTATTGGG‐3′
GAPDH reverse	5′‐CTGGAAGATGGTGATGGGATT‐3′
DR6 forward	5′‐ATCCGGAAAAGCTCGAGGAC‐3′
DR6 reverse	5′‐CTTCCCACTTGGGCTGCTAC‐3′
KIF11 forward	5′‐GCCCCAAATGTGAAAGCATT‐3′
KIF11 reverse	5′‐CTAAAGTGGGCTTTTTGTGAACTCT‐3′
TRAF4 forward	5′‐CCACCGTTTCTGCGATACCT‐3′
TRAF4 reverse	5′‐GTCTGGGTAGATCTTGGCATAG‐3′
shDR6‐1 forward	5′‐GCAGCCTATCTTTGATGACAT‐3′
shDR6‐1 reverse	5′‐ATGTCATCAAAGATAGGCTGC‐3′
shDR6‐2 forward	5′‐CCAACGCGAAACTTGAGAATT‐3′
shDR6‐2 reverse	5′‐AATTCTCAAGTTTCGCGTTGG‐3′
DR6 signal peptide forward	5′‐CTAGCTAGCGCCACCATGGGGACCTCTCCGAGCAG‐3′
DR6 signal peptide reverse	5′‐AGCTGTGGTGGTGCTAAGGAATCCAAGCAGGAGAA‐3′
3×flag forward	5′‐GACTACAAAGACCATGACGGTGATTA‐3′
3×flag reverse	5′‐CTTGTCATCGTCATCCTTGTAGTCGATGTC‐3′
DR6 (without signal peptide) forward	5′‐CAGCCAGAACAGAAGGCCTCGAATCTCATTGGCAC‐3′
DR6 (without signal peptide) reverse	5′‐GCTCTAGATTACTACAGCAGGTCAGGAAG‐3′
Fusion sequence (DR6 signal peptide and 3×flag) forward	5′‐CTAGCTAGCGCCACCATGGGGACCTCTCCGAGCAG‐3′
	5′‐TCCTTAGCACCACCACAGCTGACTACAAAGACCATGAC‐3′
Fusion sequence (DR6 signal peptide and 3×flag) reverse	5′‐CCGTCATGGTCTTTGTAGTCAGCTGTGGTGGTGCTAAG‐3′
	5′‐CTTGTCATCGTCATCCTTGTAGTCGATGTCATGA‐3′
Fusion sequence (DR6 signal peptide and 3×flag and DR6) forward	5′‐CTAGCTAGCGCCACCATGGGGACCTCTCCGAGCAG‐3′
	5′‐ACAAGGATGACGATGACAAGCAGCCAGAACAGAAGGCC‐3′
Fusion sequence (DR6 signal peptide and 3×flag and DR6) reverse	5′‐GAGGCCTTCTGTTCTGGCTGCTTGTCATCGTCATCCTTG‐3′
	5′‐GCTCTAGATTACTACAGCAGGTCAGGAAGA‐3′
KIF11 siRNA sense	5′‐UCGAGAAUCUAAACUAACU‐3′
KIF11 siRNA antisense	5′‐AGUUAGUUUAGAUUCUCGA‐3′
TRAF4 siRNA sense	5′‐GCACTAAGGAGTTCGTCTT‐3′
TRAF4 siRNA antisense	5′‐AAGACGAACUCCUUAGUGC‐3′

GAPDH, glyceraldehyde‐3‐phosphate dehydrogenase; KIF11, kinesin family member 11; shDR6‐1, short hairpin DR6‐1; shDR6‐2, short hairpin DR6‐2; TRAF4, TNF receptor‐associated factor 4.

### Patients and specimens

The First Affiliated Hospital of Harbin Medical University provided the fresh OVCA tissues and normal ovarian epithelial tissue. Patients provided informed consent. Institutional Review Board of Institute of Basic Medical Sciences, Chinese Academy of Medical Sciences approved the current study. The classifications of cancer stage and grade were made according to the International Federation of Gynecology and Obstetrics criteria 13.

### Reverse transcription‐quantitative PCR

TRIzol (Invitrogen) was used to purify total RNA according to the manufacturer's protocol. The cDNA was obtained from RNA by using M‐MLV Reverse Transcriptase (Promega, Madison, WI, USA). The expression of target RNAs was measured by qPCR with SYBR Green PCR Master Mix (Thermo Fisher Scientific, Waltham, MA, USA) on a StepOnePlus system (Applied Biosystems). Sangon Biotech (Shanghai, China) produced the primers, which were designed using Primer Premier 5.0. The thermocycling conditions for qPCR included initial denaturation (5 min at 95 °C), followed by 40 cycles (15 s at 95 °C, 30 s at 60 °C, and 30 s at 72 °C) and extension (7 min at 72 °C). The relative gene expression was calculated using the 2^−ΔΔ*C*^_T_ method, and the internal control was glyceraldehyde 3‐phosphate dehydrogenase (GAPDH). The primer sequences are shown in Table 1.

### Western blot analysis

Total protein was obtained after cells were lysed using M‐PER Mammalian Protein Extraction Reagent (Thermo Fisher Scientific) with protease inhibitor cocktail (Sigma, St Louis, MO, USA). After the protein was denatured, equal amounts of protein were loaded in each lane for SDS/PAGE, and then, the protein was transferred onto PVDF membranes. The membranes were blocked using Tris‐buffered saline (10 mM Tris, 150 mM NaCl) with 0.05% Tween 20 (TBST) with 5% nonfat milk for 120 min, then incubated separately with primary antibodies against matrix metalloprotease (MMP) 2 (polyclonal, rabbit anti‐human, cat. no. 4022), MMP9 (polyclonal, rabbit anti‐human, cat. no. 3852), E‐cadherin (monoclonal, rabbit anti‐human, cat. no. 8834), phosphoinositide‐3‐kinase (PI3K; polyclonal, rabbit anti‐human, cat. no. 3811), AKT (polyclonal, rabbit anti‐human, cat. no. 9272), phospho (p)‐ AKT (monoclonal, rabbit anti‐human, cat. no. 5012), mitogen‐activated protein kinase kinase (MEK; monoclonal, mouse anti‐human, cat. no. 4694), p‐MEK (monoclonal, rabbit anti‐human, cat. no. 9154), extracellular signal‐regulated kinase (ERK; polyclonal, rabbit anti‐human, cat. no. 4695), p‐ERK (monoclonal, rabbit anti‐human, cat. no. 8544), KIF11 (monoclonal, rabbit anti‐human, cat. no. 14404), and TRAF4 (monoclonal, rabbit anti‐human, cat. no. 18527). The antibodies were purchased from Cell Signaling Technology (Danvers, MA, USA; all 1 : 1000). Additionally, incubation with primary antibody against DR6 (monoclonal, rabbit anti‐human, cat. no. 3216R‐100; Biovision, Milpitas, CA, USA; 1 : 1000) and FLAG (monoclonal, mouse anti‐human, cat. no. M185‐3L; MBL, Nagoya, Japan; 1 : 500) dissolved in 5% nonfat milk in TBST overnight at 4 °C was performed separately. Human GAPDH antibody (monoclonal, mouse anti‐human, cat. no. KC‐5G4; KangChen Biotech, Shanghai, China; 1 : 5000) was used as an internal reference. Immunoblots were quantified using imagej software (developed by National Institutes of Health, Bethesda, MD, USA).

### Plasmid generation

The full‐length KIF11 or TRAF4 cDNA sequence was amplified from the total RNA of SKOV3 cells by RT‐PCR using the primers listed in Table 1 and inserted at EcoRI/XhoI sites of the pcDNA3.1(+) to generate pcDNA3.1‐KIF11 and pcDNA3.1‐TRAF4 overexpression plasmid.

### Lentivirus infection and construction of stable DR6‐knockdown cell lines

Short hairpin RNAs (shRNAs) targeting DR6 were inserted into pLL3.7 vectors. The targeting sequences are listed in Table 1. Lentiviruses were obtained by cotransfecting the recombinant pLL3.7 vectors and pRSV‐Rev, pMDLg/pRRE, and pMD2.G packaging vectors. Lentiviruses with infection were obtained at 48 and 72 h after transfection. The viruses were filtered with 0.45 μm poly(vinylidene) difluoride (PVDF) filters. Viruses were collected by ultracentrifugation (40 min at 80 000 ***g***). The virus precipitate was stored in RPMI 1640 at −80 °C. Infectious titers were determined by counting the number of GFP‐expressing colonies. The virus and polybrene (10 μg·mL^−1^) (Sigma‐Aldrich) were added to the cells. After 24 h, the supernatant was discarded and fresh RPMI 1640 was added in. Cells infected with virus were identified by western blot analysis at 96 h. Then flow cytometry (BD FAS Aria III Cell Sorter, Beckman Coulter, Miami Lakes, FL, USA) was used to identify and collect SKOV3 cells with GFP.

### Cancer cell migration assay

Transwell insert chambers with 8‐μm porous membranes (Corning Inc., Corning, NY, USA) were used for motility assays. Approximately 2 × 10^5^ cells without FBS were added to the upper chamber, and medium with 10% FBS was added to the lower chamber. The cells were cultured for 24 h. The cells on the upper surface of the upper chamber were scraped off using a cotton bud, and the chamber was rinsed with phosphate buffer. Then, the cells on the lower surface of the chamber were soaked in 4% paraformaldehyde. Once the surface was dry, the cells were dyed with 1% crystal violet for 2 min. The migrating cells were those on the lower surface of the chamber. Migrating cells were counted at ×100 or ×200 magnification in eight fields of view.

### Immunofluorescence experiments

The cells were washed with phosphate buffer and fixed with 4% paraformaldehyde for 20 min. Then, the cells were incubated for 10 min with 0.2% Triton X‐100 to change the permeability and washed three times with 0.01 m phosphate buffer (5 min per wash). Next, the cells were soaked in 1% BSA at 37 °C for 20 min and then incubated separately with primary antibody [anti‐FLAG (monoclonal, mouse anti‐human, cat. no. M185‐3L; MBL; 1 : 500) and anti‐KIF11 (monoclonal, rabbit anti‐human, cat. no. 14404; Cell Signaling Technology; 1 : 500)] diluted with 1% BSA, at room temperature for 1 h. The cells were incubated separately with secondary antibody (Cell Signaling Technology, cat. no. 8890, cat. no. 4412, 1 : 200) and Hoechst (1 : 200) for 1 h after being washed with 0.05% Tween‐20. Next, the cells were washed three times with 0.05% Tween‐20 (5 min per wash), then with 0.01 m phosphate buffer for 5 min. The cells were observed under a fluorescence microscope.

### Immunoprecipitation

The signal peptide of DR6, 3×Flag, and DR6 sequences was fused by PCR. The primers are listed in Table 1. The fused sequence was cloned into pcDNA3.1. HEK293T cells were transiently transfected with pcDNA3.1‐Flag‐DR6 using Lipofectamine 2000 according to the manufacturer's instructions. Then, DR6 expression was checked by western blotting at 48 h after transfection. When the immunoprecipitation experiment was performed, the cells transfected with pcDNA3.1‐Flag‐DR6 in 10‐cm culture dishes were collected and washed three times with phosphate buffer. The cells were subjected to a 30‐min lysis with buffer (pH 7.5, 150 mm NaCl, 20 mm Tris/HCl, 10% glycerol, 1% Triton X‐100, 2 mm EDTA) including protease inhibitors (Roche, Indianapolis, IN, USA). The mixture was centrifuged at 13 000 ***g*** for 30 min at 4 °C and then used for immunoprecipitation (IP) with FLAG antibody or DR6 antibody for 8 h on ice. Then, protein A agarose beads were added to the mixture and shaken for 6 h at 4 °C. The immunoprecipitates were washed five times with phosphate buffer. The mixture was used for SDS/PAGE (5% acrylamide for the spacer gel and 12% acrylamide for the separation gel). Then, the proteins were silver stained or transferred to a PVDF membrane, which was subjected to chemiluminescence and exposure.

### Glutathione *S*‐transferase pull‐down

Approximately 600 μg lysates of HEK‐293T cells (Cell Culture Center, Chinese Academy of Medical Sciences, Beijing, China) transfected with Flag‐DR6 were used for glutathione *S*‐transferase (GST) pull‐down assay *in vitro*. The lysis buffer was composed of 50 mm Tris/HCl (pH 7.4), 150 mm NaCl, 1% NP‐40, 1 mm EDTA, and protease inhibitor cocktail. The lysates were blended with 6 μg of GST–KIF11 or GST. These mixtures were combined with glutathione/Sepharose beads for 4 h at 4 °C and washed 10 times with buffer. The mixtures were run on 12% SDS/PAGE and then incubated with anti‐FLAG (monoclonal, mouse anti‐human, cat. no. M185‐3L; MBL; 1 : 200) and anti‐GST antibodies (monoclonal, mouse anti‐human, cat. no. 2625, Cell Signaling Technology; 1 : 200) separately. The antibodies were diluted with 1% BSA.

### Statistical analysis

Statistical analysis was performed using spss version 17.0 software (SPSS, Inc., Chicago, IL, USA). One‐way ANOVA and Student's *t* test were used for data analysis. Two‐sided *P *< 0.05 was considered to indicate a statistically significant difference.

## Results

### Death receptor 6 is upregulated in OVCA

**Figure 1:**
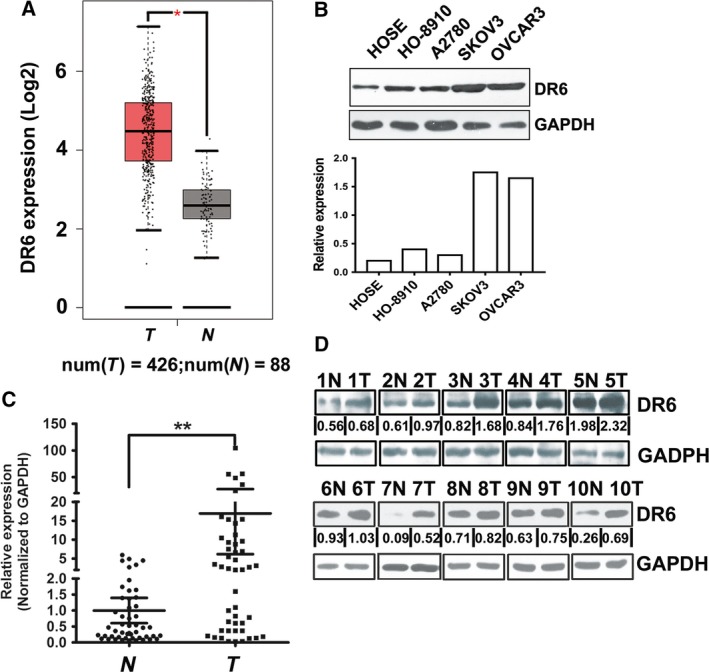
Death receptor 6 is expressed highly in ovarian cancer. (A) DR6 expression in human OVCA based on analysis of TCGA database. (B) Representative western blot showing DR6 protein levels in HOSE cells and the four indicated OVCA cell lines. (C) DR6 expression was analyzed by qRT‐qPCR in OVCA tissues and normal tissues. SD is represented with bars (*N* = 50, *T* = 50). (D) DR6 expression in normal tissues and OVCA tissues was evaluated by western blot analysis. *N* represents normal tissues, and *T* represents tumor tissues. The loading control was GAPDH. **P < 0.01.

On analysis of The Cancer Genome Atlas (TCGA) OVCA and normal tissue RNA sequencing data (http://gepia.cancer-pku.cn/index.html) 14, the expression level of DR6 was higher in OVCA than in normal tissues (Fig. 1A). We subsequently analyzed the expression level of DR6 in OVCA cells by western blot analysis. As shown in Fig. 1B, compared with normal human ovarian surface epithelial (HOSE) cells, DR6 was significantly upregulated in the four OVCA cell lines (SKOV3, HO‐8910, A2780, and OVCAR3). Total RNA and protein were isolated from OVCA tissues and from matched adjacent normal tissues to detect the expression level of DR6. The expression of DR6 was analyzed by quantitative PCR and western blot. The results indicated that the mRNA and protein expression levels of DR6 were higher in OVCA samples than they were in the matched adjacent normal samples (Fig. 1C,D).

### DR6 promotes OVCA cell migration *in vitro*

**Figure 2:**
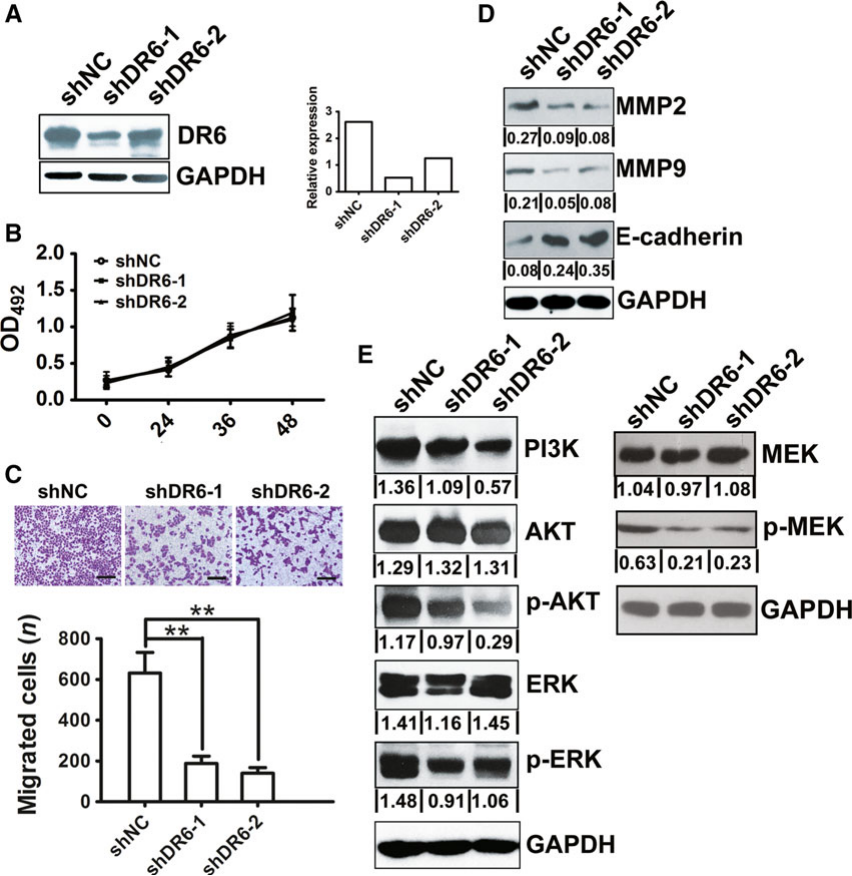
Death receptor 6 promoted migration in OVCA cells. (A) DR6 expression in SKOV3 cells with stable DR6 knockdown was evaluated by western blot analysis. (B) MTS assay assessed the proliferation of SKOV3 cells with stable DR6 knockdown. (C) The effect of DR6 knockdown on migration in SKOV3 cells was investigated by transwell assay. Scale bar = 100μm. (D) Evaluation of the effect of DR6 knockdown in SKOV3 cells on MMP2, MMP9, and E‐cadherin expression by western blot analysis. (E) The analysis of the effect of DR6 knockdown in SKOV3 cells on PI3K/AKT and MAPK/ERK pathway regulation by western blot analysis. Data are presented as mean ± SD (*n* = 8). **P < 0.01 (Student's *t* test).

It was shown that among the OVCA cells assessed, DR6 expression was highest in SKOV3 cells (Fig. 1B). Thus, in subsequent assays, the roles of DR6 were investigated in SKOV3 cells. To study the potential role of DR6 in cell growth, the proliferation of SKOV3 cell lines transfected with DR6 shRNA vectors was analyzed by 3‐(4,5‐dimethylthiazol‐2‐yl)‐5‐(3‐carboxymethoxyphenyl)‐2‐(4‐sulfophenyl)‐2*H*‐tetrazolium (MTS) assay. As shown in Fig. 2A, DR6 shRNA induced a corresponding downregulation in DR6. The MTS assays showed that DR6 knockdown did not change the viability of OVCA cells (Fig. 2B). Next, a transwell assay was performed to detect cell migration ability following the knockdown of DR6 in SKOV3 cells. After transfection with shRNA to decrease endogenous DR6 expression, the migration of the SKOV3 cells was markedly reduced (Fig. 2C). Furthermore, the migration‐associated protein expression in SKOV3 cells was examined by western blot analysis. The results indicated that when the expression of DR6 was inhibited, the expression of MMP2 and MMP9 was inhibited, but the expression of E‐cadherin was enhanced (Fig. 2D).

The PI3K/AKT and mitogen‐activated protein kinase (MAPK) /ERK signaling pathways have been widely reported to be among the most important signaling pathways that participate in a regulatory network during cell migration in various cancers 14, 15, 16, 17. The expression level of PI3K, p‐AKT, p‐MEK, and p‐ERK was evaluated by western blot analysis. The results showed that DR6 shRNA led to an obvious decrease in PI3K, p‐AKT, p‐MEK, and p‐ERK expression in SKOV3 cells, compared with the vector control groups. This finding indicated that the activation of the PI3K/AKT or MAPK/ERK pathway might participate in the effect of DR6 on OVCA cell migration (Fig. 2E).

### Identification of DR6‐interacting proteins by coimmunoprecipitation–mass spectrometry

**Figure 3:**
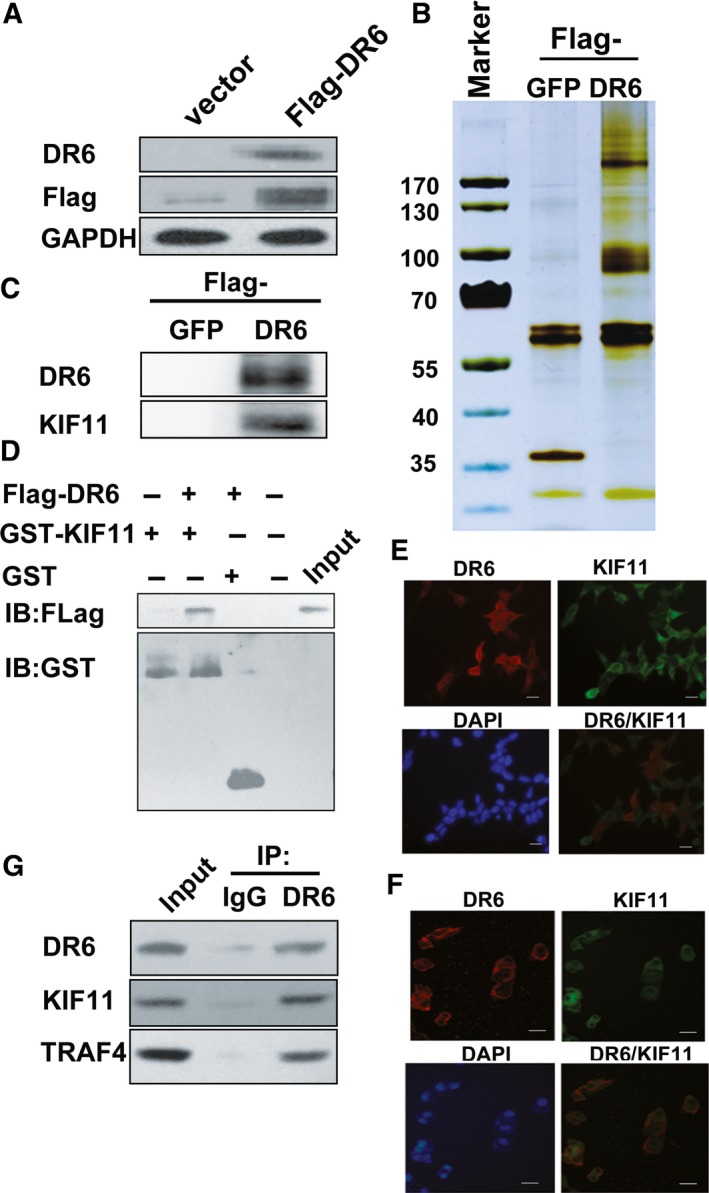
Co‐IP–MS identified the DR6‐interacting proteins. (A) Identification of DR6 expression in HEK‐293T/Flag‐DR6 cells with western blot analysis. The loading control was GAPDH. (B) Proteins identified by silver staining were extracted from HEK‐293T/Flag‐DR6 cell lysates by co‐IP. (C) Co‐IP of DR6 transfected and KIF11 in SKOV3 cells. Flag‐DR6 was transfected into in SKOV3 cells. Anti‐Flag antibodies immunoprecipitated cell lysates after a 48‐h transfection. The detection of IB was conducted with anti‐KIF11 and anti‐DR6 antibodies. (D) Direct interaction of DR6 and KIF11 was analyzed by GST pull‐down analysis. The anti‐GST and anti‐Flag antibodies detected the input and pull‐down samples. (E,F) Colocalization of DR6 and KIF11 in HEK‐293T cells (E) and SKOV3 cells (F). Immunofluorescence analysis was performed. The nuclei were stained with 4′,6‐diamidino‐2‐phenylindole (DAPI). Scale bar = 10 μm. (G) Co‐IP of DR6, KIF11, and TRAF4 in SKOV3 cells. DR6 antibody or IgG antibody immunoprecipitated the lysates and IB of DR6, KIF11, or TRAF4 antibodies was performed.

To confirm the molecular mechanism of DR6 in OVCA migration, we used the co‐IP–mass spectrometry (MS) method to find DR6‐interacting proteins in HEK‐293T cells. Cells expressing Flag‐DR6 recombinant protein and cells expressing Flag‐GFP recombinant protein were cultured. Flag‐DR6 expression was identified by Flag antibody (Fig. 3A). Flag agarose purified cellular lysate, and the mixture (including anti‐Flag antibody, Flag‐DR6 and interaction protein with DR6) obtained was run on an SDS/PAGE gel and stained with silver (Fig. 3B). DR6 co‐purified with several proteins by MS analysis, among which the content of KIF11 was highest. To confirm the interaction between KIF11 and DR6, total protein from SKOV3 cells transfected Flag‐DR6 was extracted, and co‐IP experiments were performed with FLAG antibodies against proteins. IP with an antibody against FLAG followed by immunoblotting (IB) with an antibody against KIF11 demonstrated that DR6 co‐immunoprecipitated with KIF11 (Fig. 3C). Furthermore, we found that DR6 and KIF11 could interact directly in a GST pull‐down assay (Fig. 3D). DR6 and KIF11 also localized together on the cytomembrane of HEK‐293T and SKOV3 cells when analyzed by immunofluorescence (Fig. 3E,F). It was reported that TRAF4 was identified as an interacting protein of KIF11 in a previous MS analysis 18. Therefore, we subsequently tested whether DR6 could bind TRAF4 using co‐IP. As shown in Fig. 3G, IP with an antibody against DR6 and IB with antibodies against TRAF4 and KIF11 revealed that TRAF4 and KIF11 co‐immunoprecipitated with DR6 simultaneously.

### DR6 functions through its association with KIF11 and TRAF4

**Figure 4:**
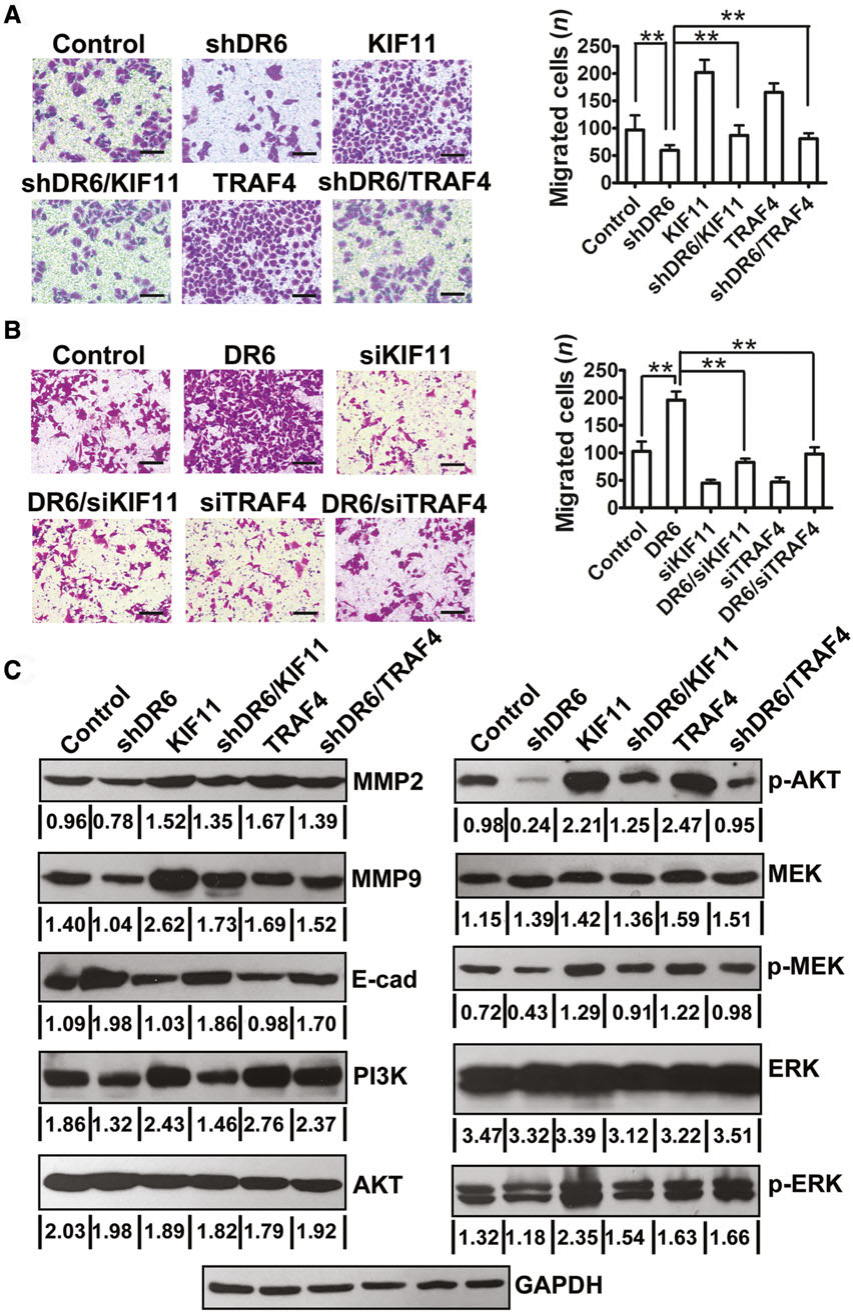
DR6 functions through its association with KIF11 and TRAF4. (A) Overexpression of KIF11 or TRAF4 reversed the DR6 knockdown‐mediated reduction in SKOV3 cell migration. Scale bar = 200μm. (B) Knocking down of KIF11 or TRAF4 reversed the DR6 overexpression‐mediated increase in SKOV3 cell migration. Scale bar = 200μm. (C) Western blot analysis of indicated protein expression in SKOV3 cells. Data are presented as mean ± SD (*n* = 8). **P < 0.01 (Student's *t* test).

Whether the function of DR6 was based on the association of DR6 with KIF11 and TRAF4 was evaluated using KIF11 or TRAF4 overexpression (Fig. S1) in SKOV3 cells. As shown in Fig. 4A, the overexpression of KIF11 or TRAF4 promoted SKOV3 cell migration. Next, using a rescue experiment, we evaluated whether the DR6‐mediated promotion of SKOV3 cell migration depended on its association with KIF11 and TRAF4. As shown in Fig. 4A, the inhibition of cell migration ability in the DR6‐knockdown SKOV3 cells was rescued by KIF11 or TRAF4 overexpression. Accordingly, the promotion of cell migration ability in the DR6‐overexpression SKOV3 cells was rescued by KIF11 siRNA or TRAF4 siRNA (Fig. S2) knockdown (Fig. 4B). The expression of DR6, KIF11, and TRAF4 was evaluated in all the different experimental conditions shown in Figs S3 and S4. Furthermore, the migration‐associated protein expression of PI3K/AKT and MAPK/ERK signaling pathways in the rescue experiment was also examined by western blot analysis. The results indicated that the expression variation of MMP2, MMP9, and E‐cadherin induced by DR6 knockdown was also rescued by TRAF4 or KIF11 overexpression (Fig. 4C), and decreased activation of PI3K, p‐AKT, and p‐MEK with DR6 shRNA was rescued by TRAF4 or KIF11 overexpression (Fig. 4C). However, the activation of p‐ERK was rescued by KIF11 overexpression but not by TRAF4 overexpression; this indicated that TRAF4 might be partly involved in another signalling pathway transduced by MEK activation.

**Figure 5:**
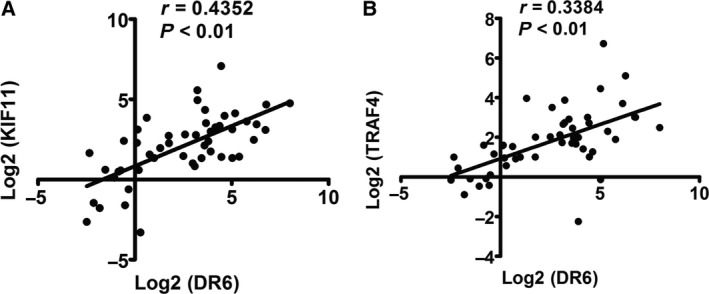
The correlation between DR6 and KIF11/TRAF4 expression in OVCA. (A) The correlation between DR6 and KIF11 expression in OVCA tissues (*n* = 50). (B) The correlation between DR6 and TRAF4 expression in OVCA tissues (*n* = 50).

Furthermore, we compared the expression of KIF11 and TRAF4 with that of DR6 and found that the mRNA expression of KIF11 was correlated with DR6 expression in OVCA tissues (Fig. 5A; *r* = 0.4352, *P *< 0.01). Additionally, TRAF4 mRNA expression was positively correlated with DR6 expression in the OVCA tissues (Fig. 5B; *r* = 0.3384, *P *< 0.01). This concordant correlation of TRAF4 and KIF11 expression with that of DR6 suggested that a DR6–TRAF4–KIF11 complex may play an important role in OVCA migration.

## Discussion

Although DR6 is expressed highly in many cancer types, its role and underlying molecular mechanisms in cancer progression remain unclear. It has been found previously that DR6 level in the serum increases in patients with late‐stage OVCA 11. Through analysis of TCGA database, we also identified DR6 overexpression in OVCA. Therefore, we aimed to investigate the function of DR6 in OVCA.

Several previous reports have shown that DR6 is closely associated with vascular endothelial cells. For instance, the number of microvessels expressing DR6 was obviously more in hens with early‐stage OVCA than in hens with normal ovaries and was most in hens with late‐stage OVCA 19. Additionally, it was reported that DR6, as a confirmed tumor vascular marker, could also function as a serum biomarker in OVCA vasculature 11. Tam *et al*. found in both zebrafish and mice that DR6 and TROY could regulate angiogenesis of the central nervous system. The interaction between DR6 and TROY was genetic and physical. And vascular endothelial growth factor mediated by c‐Jun N‐terminal kinase (JNK) activation was required for the interaction 5. Strilic *et al*. reported that endothelial cell necroptosis induced by DR6 in tumor cells promoted cancer metastasis 12. We also previously demonstrated that tumor angiogenesis of B16 required DR6, which was via the nuclear factor‐κB, P38 MAPK, and signal transducer and activator of transcription 3 (STAT3) pathways 20. Based on these reports, DR6 can be concluded to be vital in the function of endothelial cells. In this study, our data showed that DR6 enhanced ovarian carcinoma cell migration ability via the MAPK/ERK and PI3K/AKT pathways. Therefore, not only DR6 expressed on endothelial cells involved in cancer metastasis, but DR6 expression on cancer cells may also promote cancer metastasis.

To investigate the molecular signaling pathway of DR6 in cancer migration, a co‐IP–MS strategy was conducted. KIF11 was confirmed to interact with DR6 by co‐IP and GST pull‐down assay. KIF11 is an important kinesin and exerts an obvious effect in the polarization of spindle apparatus in mitosis 21. This may explain the overexpression of KIF11 in some tumors and leukemias compared with the normal sample 22, 23. The regulation of mitosis, migration, and intracellular transport by KIF11 occurs via interactions with microtubules 21, 24, 25. Using MS analysis, a previous study identified that TRAF4 was an interacting protein of KIF11 18. The TRAF family was originally identified as a group of signal adaptors that interact directly with cytoplasmic parts of members of the TNF receptor superfamily. In human cancers, TRAF4 was overexpressed, and the gene was amplified 26, 27, 28. Additionally, it was found that TRAF4 could enhance cell growth and migration in certain tumor cells 29, 30. Thus, we also determined the relationship between DR6 and TRAF4. As shown by our results, IP with an antibody developed against DR6 and IB with antibodies developed against TRAF4 and KIF11 indicated that TRAF4 and KIF11 co‐immunoprecipitated with DR6 simultaneously; this co‐IP suggests that a DR6–TRAF4–KIF11 complex may have a crucial function in OVCA migration. However, the formation of this protein complex requires further investigation. The differential effect of shTRAF4 and shKIF11 on p‐ERK but not on MMP2, MMP9, or PI3K indicated that TRAF4 might be partly involved in other signal pathways transduced by MEK activation. MEK pathway is normally coupled to ERK. However, some studies showed that ERK could be activated without MEK activation. Simard *et al*. 31 showed that in human neutrophils stimulated with cytokines or Toll‐like receptor ligands, MEK and ERK are activated independently of each other. This coupling of MEK and ERK was not observed. Aksamitiene *et al*. 32 confirmed the existence of a dynamic MEK‐independent compensatory circuit for ERK activation in T47D cells that is induced by epidermal growth factor and other ligands of ErbB family receptors. MEK‐independent ERK activation depends on PDZ‐binding kinase/T‐LAK‐cell‐originated protein kinase (PBK/TOPK). Consequently, the role of DR6 in OVCA related to the uncoupling of the MEK and ERK pathway will be addressed in our further research.

## Conclusion

A novel function of DR6 in promoting cell migration was identified. The present discovery showed that DR6 has a significant effect on ovarian malignancy by interacting with TRAF4 and KIF11, and that targeting DR6 might be an important therapeutic strategy in the treatment of OVCA.

## Author contributions

BS wrote the article and performed experiments. JB performed cell experiments. YL performed cell experiments. JS directed the project.

## Supporting information


**Fig. S1.** Western blot analysis of expression of indicated proteins in SKOV3 cells transfected with pcDNA3.1‐KIF11 or pcDNA3.1‐TRAF4.
**Fig. S2.** Western blot analysis of expression of indicated proteins in SKOV3 cells transfected with KIF11 siRNA or TRAF4 siRNA.
**Figs S3 and S4.** The expression of DR6, KIF11 and TRAF4 evaluated in all the different experimental conditions.
