# Supplementary figures and images for: Death receptor 6 promotes ovarian cancer cell migration through KIF11
Source: FEBS Open Bio. 2018 Aug 7;8(9):1497–507. doi: 10.1002/2211-5463.12492 (PMC6120224; doi:10.1002/2211-5463.12492)

Figure S1.

Figure S2.


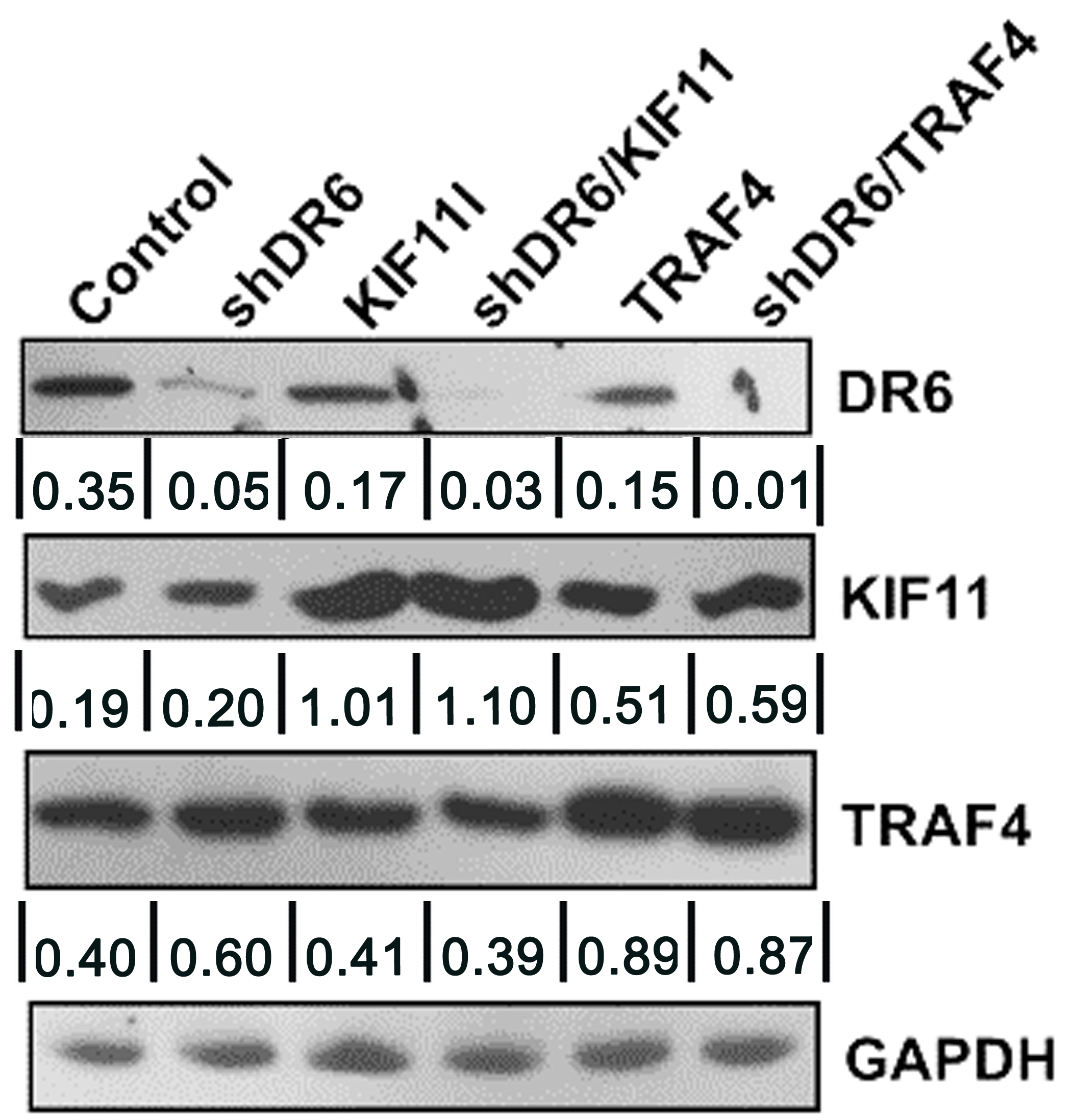


Figure S3.


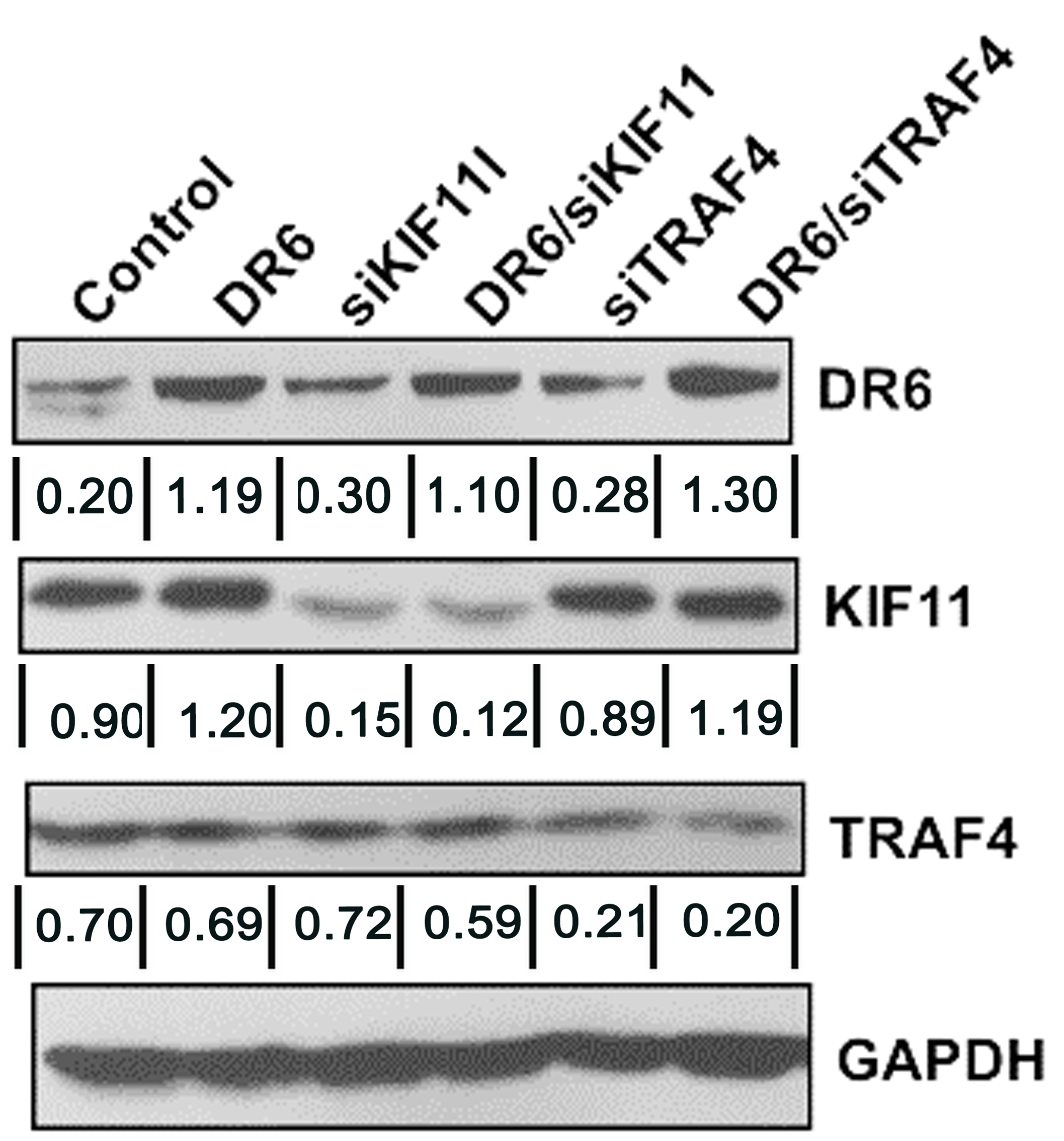


Figure S4.

Supplement: Supplementary file 1 — Fig. S1. Western blot analysis of expression of indicated proteins in SKOV3 cells transfected with pcDNA3.1‐KIF11 or pcDNA3.1‐TRAF4. Fig. S2. Western blot analysis of expression of indicated proteins in SKOV3 cells transfected with KIF11 siRNA or TRAF4 siRNA. Figs S3 and S4. The expression of DR6, KIF11 and TRAF4 evaluated in all the different experimental conditions. [file FEB4-8-1497-s001.doc]
